# Research on Biomimetic Models and Nanomechanical Behaviour of Membranous Wings of Chinese Bee *Apis cerana cerana* Fabricius

**DOI:** 10.1155/2018/2014307

**Published:** 2018-02-19

**Authors:** Yanru Zhao, Dongsheng Wang, Jin Tong, Jiyu Sun, Jin Zhang

**Affiliations:** ^1^The College of Mechanical and Power Engineering, Henan Polytechnic University, Jiaozuo 454000, China; ^2^The Key Laboratory of Bionic Engineering, Ministry of Education and the College of Biological and Agricultural Engineering, Jilin University, Nanling Campus, Changchun 130022, China; ^3^United Automotive Electronic Systems Co. Ltd., Shanghai 200051, China

## Abstract

The structures combining the veins and membranes of membranous wings of the Chinese bee *Apis cerana cerana* Fabricius into a whole have excellent load-resisting capacity. The membranous wings of Chinese bees were taken as research objects and the mechanical properties of a biomimetic model of membranous wings as targets. In order to understand and learn from the biosystem and then make technical innovation, the membranous wings of Chinese bees were simulated and analysed with reverse engineering and finite element method. The deformations and stress states of the finite element model of membranous wings were researched under the concentrated force, uniform load, and torque. It was found that the whole model deforms evenly and there are no unusual deformations arising. The displacements and deformations are small and transform uniformly. It was indicated that the veins and membranes combine well into a whole to transmit loads effectively, which illustrates the membranous wings of Chinese bees having excellent integral mechanical behaviour and structure stiffness. The realization of structure models of the membranous wings of Chinese bees and analysis of the relativity of structures and performances or functions will provide an inspiration for designing biomimetic thin-film materials with superior load-bearing capacity.

## 1. Introduction

Insects have developed their flight capacities of individual character during the long process of nature selection. The membranous wings of an insect are organs for flight behaviour, the weight of which is only 1-2% of its whole weight [1, 2]. The lifting force produced by insect membranous wings is about ten times higher than that of airfoil with the same area. Insects are magnificent in flight, and the flying behaviours include flapping flight, glide, hover, changing flying directions rapidly, and flight backward [3].

The research on biological membrane has attracted widespread attention due to insect membranous wings with specific structures and superior performances. The biological membrane is a new field formed in the research of international high-tech materials in the 1990s. The research on biological membrane is very active in developed countries such as America, Japan, and Germany; moreover, it is of great importance in reforming traditional industries, for example, agriculture, building material, aviation, spaceflight, military industry, automobile, and cutting tool [4–6]. The biomimetic composite membrane has a very broad market prospect and important strategic significance, can be used on the airfoil of micro aerial vehicle and the bionic coating of military equipment, and can also be used as environmental protection material to replace harmful plastic products, for example, agricultural film [7]. The research on biological membrane has important meaning on bionics to biomimetic composite membrane and has extensive application prospect on the fields of information technology, biotechnology, and new energy technology.

The research imitating insect membranous wings has been a hotspot at home and abroad. The model design imitating insect membranous wings has practical significance. The insect species is more and the forms of membranous wings are complicated and varied, which provides abundant natural sources for the research on biomimetic thin-film materials and biomimetic 2-dimensional composite materials [8, 9]. There are many factors affecting the model imitating insect membranous wings, such as the material property of veins and membranes, distribution and quantity of veins, and appearance of membranous wings [10–12]. The materials that are alike to insect membranous wings are less, so the appearance of membranous wings and distribution of veins are excellent reference for the biomimetic research before ideal materials can be found.

## 2. Materials and Methods

### 2.1. Sample Preparation and Test Equipment

**Figure 1:**
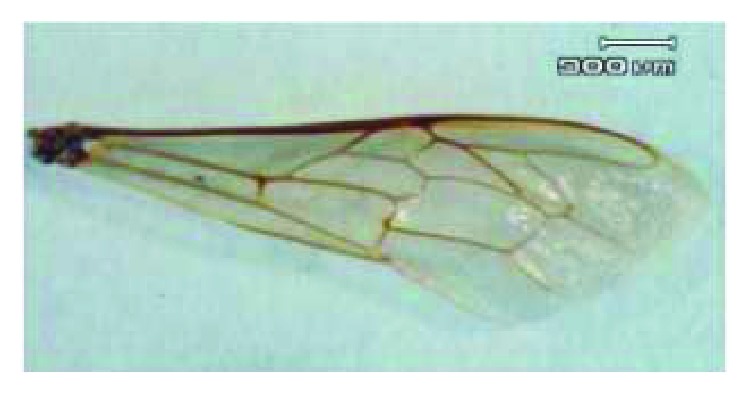
Stereoscopy photograph of the right forewing of a Chinese bee.

Living Chinese bees, *Apis cerana cerana* Fabricius (Hymenoptera, Aculeata, Apoidea, Apidae), were gathered. The Chinese bees were anaesthetized, and then their membranous wings were cut off their bodies within 12 hours so that the in vivo structures can be reserved. The membranous wings were cleaned with distilled water and then were air-dried. The right forewings of Chinese bees were selected as research objects, because the right forewings with large areas are the main wings to bear loads in flight and have representativeness. The mass and morphological parameters of Chinese bees were measured by an electronic analytical balance (FA2004), a stereomicroscope (SZX12), and its image analysis system (OLYCIA™ M3).  Figure 1 shows a stereoscopy photograph of the right forewing of the Chinese bee *Apis cerana cerana* Fabricius. Five Chinese bees were chosen randomly, and their morphological parameters were surveyed, and then the mean value was taken as the result. The mass *m* = 94.38 mg = 9.438 × 10^−5^ kg. Length of the right forewing *a* = 8.6 mm, and width of the right forewing *b* = 2.8 mm. The area of the right forewing *A* = 13.2 mm^2^.

In order to avoid the influence of reflectivity, color, and curvature difference of the membranous wings on point cloud data collection, the dye penetrant imaging agent DPT-5 was used to deal with the membranous wings. Issues such as the fixing of the membranous wings, the spraying distance, and the uniformity of the coating should be paid attention to in spraying. Make sure that the coating is as thin and uniform as possible to diminish the effects of the spray to the results due to the membranous wings forming freeform surfaces [13–15].

A 3D laser-scanning system (LSV50) developed by 3D Family Technology Co. Ltd. was adopted. The surfaces of membranous wings of Chinese bees were scanned many times. The point cloud data were obtained by adjusting the scanning step and altering the measuring angle and position. The point cloud data are the base of the model reconstruction, processing manufacturing, analog simulation, and feature analysis.

### 2.2. Setting Up of Structure Models of Membranous Wings of the Chinese Bee

The membranous wings of the Chinese bees dealt with DPT-5 were scanned by 3D laser-scanning system and were kept parallel to the scanning plane throughout the whole scanning process. The point cloud data were obtained, which inevitably introduces data error especially near sharp edges and the boundaries. The defective points of the point cloud data may cause the surface formed to deviate from the original surface; thus, the model reconstructed will be influenced. The jump points would come into being due to the change of calibration parameters of measuring equipment and the abrupt change of the measurement environment. The defective points and jump points have a great influence on the smoothness of curves in surface modeling. The sample holder and backing plate would also be scanned unavoidably in scanning, so first of all, the data unrelated to the research should be deleted during data processing.

The quality of point cloud data would become bad because of the interference by various noise. The point cloud data should be smoothed in order to eliminate or reduce the effects of noise, that is to say, let low-frequency data pass through and high-frequency noise are intercepted. The quality of a 3-dimensional model reestablished would be enhanced by data smoothing [16].

The information supplied by the parameters should remain constant, adopting a smoothing method. If the smoothness of data points of infinite nodes is considered, the offset function {*P*_*n*_} smoothed can be computed with linear superposition of the offset function {*P*_*v*_} as follows.

(1) $$\begin{array}{c} \left\{P_{v}\right\}\;\left(v=\ldots ,-1,0,1,\ldots \right),\\ P_{n}=\overset{+\infty }{\underset{v=-\infty }{\sum }}P_{v}L_{n-v}, \end{array}$$

where {*P*_*v*_} is the offset function, {*P*_*n*_} is the offset function smoothed, and {*L*_*v*_} is the weighting factor and pair series, namely, *L*_−*v*_ = *L*_*v*_.

The data {*P*_*n*_} is smoother than {*P*_*v*_}; that is, the fluctuation of new data does not exceed that of the original data. The data disposed is not only smoother than before but also the deviation could not be too large.

Using reverse-engineering software, ImageWare, the point cloud data of the membranous wings of the Chinese bees were smoothed with Gaussian filter. The weight in the specified domain of the Gaussian filter is Gaussian distribution. The average effect of the Gaussian filter is small, and the topography of the original data can be maintained well.

A large number of point cloud data would come into being when scanning the membranous wings of Chinese bees with the 3D laser-scanning system. High-density point cloud data contain massive redundant data. If the models are created with the point cloud data directly, a lot of time is needed to store and dispose the data and the whole process is uncontrollable. The point cloud data should be simplified before modeling.

The chord deviation sampling was used to reduce the point cloud data of the membranous wings of Chinese bees. The sampling of the scanning data is carried out by two parameters, the maximum deviation and maximum space in the chord deviation sampling. The maximum chord length was set, and points exceeding the extent would be deleted. The chord length between each point sampled and its adjacent points is within maximum deviation, and the space among points reserved is no more than the maximum space. In the chord deviation sampling, the characteristic points of surfaces reformed can be held well by identifying the curvature changes of data points so that the feature points and boundary points in the high curvature-variation region are persisted.

In reverse engineering, a common method for model reconstruction is to fit the point cloud data into spline curves with interpolation or approximation and the surfaces are accomplished with modeling tools such as sweep, blend, laying off curve, and four-side surface, and then the whole model can be gained by extension, clipping, and transition [17].

The models of Bezier, B-Spline, and NURBS have been used widely in CAD/CAM systems. For complex curves, Bezier and B-Spline are fit to deal with flat data, but NURBS can dispose of uneven data well [18–20]. The NURBS model was adopted to reconstruct the 3-dimensional geometric models of the membranous wings of Chinese bees.

The equation of NURBS curves can be shown as follows.

(2) $$\begin{array}{c} C\left(u\right)=\frac{\sum_{i=0}^{n}N_{i,p}\left(u\right)\omega_{i}P_{i}}{\sum_{i=0}^{n}N_{i,p}\left(u\right)\omega_{i}}=\overset{n}{\underset{i=0}{\sum }}R_{i,p}\left(u\right)P_{i}, \end{array}$$

(3) $$\begin{array}{c} R_{i,p}\left(u\right)=\frac{N_{i,p}\left(u\right)\omega_{i}}{\sum_{j=0}^{n}N_{i,p}\left(u\right)\omega_{i}}, \end{array}$$

where *P_i_* is the control point; *N_i,p_*(*u*) is the B-Spline basis function of *p* order, *ω_i_* is the weighted value, and *u* is the parameter value.

The 1-dimensional parameters are extended to 2-dimensional, and then the NURBS surfaces can be obtained, namely,

(4) $$\begin{array}{c} S\left(u,v\right)=\frac{\sum_{i=0}^{m}\sum_{j=0}^{n}N_{i,p}\left(u\right)N_{j,q}\left(v\right)\omega_{ij}P_{ij}}{\sum_{r=0}^{m}\sum_{k=0}^{n}N_{r,p}\left(u\right)N_{k,q}\left(v\right)\omega_{rk}}=\overset{m}{\underset{i=0}{\sum }}\overset{n}{\underset{j=0}{\sum }}R_{i,p\text{;}j,q}\left(u,v\right)P_{ij}, \end{array}$$

(5) $$\begin{array}{c} R_{i,p\text{;}j,q}\left(u,v\right)=\frac{N_{i,p}\left(u\right)N_{j,q}\left(v\right)\omega_{ij}}{\sum_{r=0}^{m}\sum_{k=0}^{n}N_{r,p}\left(u\right)N_{k,q}\left(v\right)\omega_{rk}}, \end{array}$$

where *P_ij_* is the control point of the surface and *R*_*i*,*p*;*j*,*q*_(*u*, *v*) is the basis function of the NURBS surface.

**Figure 2:**
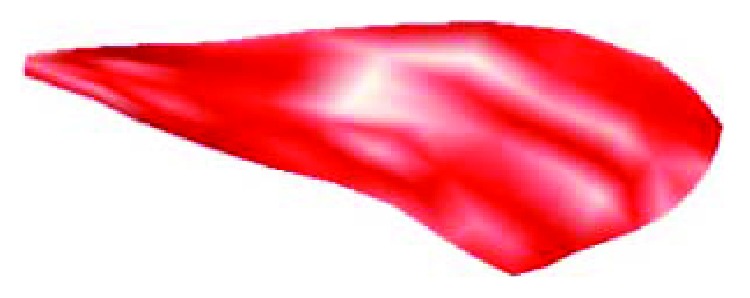
Structure model of the right forewing of a Chinese bee.

According to the appearance characters of membranous wings of Chinese bees, the boundary curves were extracted from the point cloud data by the order Circle-Select Points, and then the 3-dimensional geometry model was rebuilt with the boundary curves and the rest of the point cloud data. The control points were added to the smoothed curves. The close degree to the real curves is good with more control points, and the curves would be smooth with less control points. Because the directions of curves are different and the distributions of control points are inhomogeneous, distortion, deformation, and reductus would come into being if the curves are used to form the surfaces directly. The boundary curves extracted should be close to the real boundaries of membranous wings of Chinese bees by means of adjusting the directions of curves, controlling the control points, adding or deleting nodes, smoothing curves, and reparametrization. The complete boundary curves were constructed by connecting the boundary curves adjusted together with the order Match 2 Curves. The model of the membranous wings of Chinese bees was accomplished with the boundary curves and the mixed point cloud data, as shown in Figure 2.

The morphological characters and main distribution of veins of membranous wings of Chinese bees were reproduced accurately by the structure model, which would provide a base for the setup of an exact finite element model.

### 2.3. Setup of Finite Element Model

The finite element software ANSYS does not specify a system unit for the analytical results. In structural analysis, any a self-contained unit system can be used according to the relevant parameters of different objects. A self-contained unit system is one in which the unit dimensions can be deduced from each other; that is, the data unit's input belongs to the same unit system in use [21]. All of the units relate to length and force, so other dimensions can be deduced from length, force, and time. Taking the smallness of membranous wings of Chinese bees into account, set length unit mm, force unit kN, mass unit kg, and time unit ms.

The veins of Chinese bees are hollow and their cross sections are approximately circular which were surveyed with a transmission electron microscopy (JEM-1200EX) made in Japan. The veins are formed of tracheas thickening, acting as the frame to sustain membranous wings, and the annular veins have better flexibility. The parameters of veins and membranes were gained. The parameters of veins applied in finite element models were divided into two classes approximately: the external diameter of the first class veins is *R*_1_ = 130 *μ*m = 0.13 mm and the wall thickness *h*_1_ = 30 *μ*m = 0.03 mm; the second class is *R*_2_ = 60 *μ*m = 0.06 mm and the wall thickness *h*_2_ = 15 *μ*m = 0.015 mm. The thickness of membranes is *h* = 1.5 *μ*m = 0.0015 mm.

The material properties were obtained by a nanomechanical testing system, alias the nanoindenter (TriboIndenter) produced by Hysitron company, USA [22–25]. The maximum and minimum indenting forces used for tests were 30 mN and 100 nN, respectively; the load resolution is less than 1 nN and the step size of lengthway displacement is 13 nm. A Berkovich tip was used for determining the nanomechanical parameters of the wings. This kind of tip is often applied as a standard tip for nanoindentation tests.

**Figure 3:**
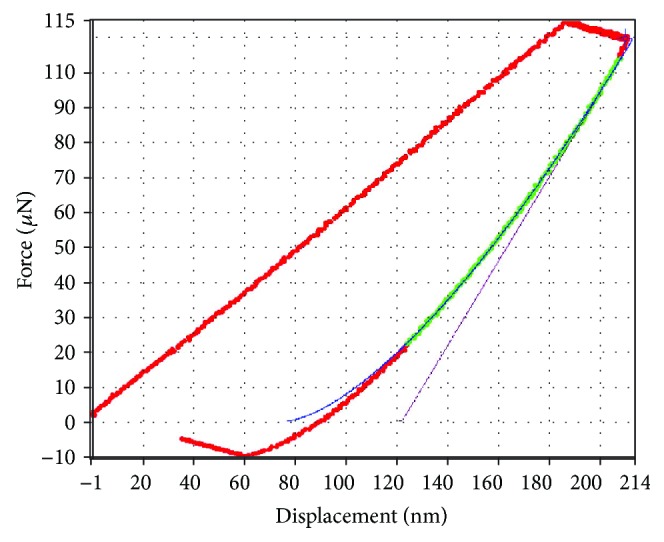
The force–displacement curve.

A force–displacement curve can be obtained in real time and is shown in Figure 3 during the course of examining the nanomechanical parameters of the material with the nanoindenter. The nanohardness, the elastic modulus, the friction coefficient, the fracture stiffness, and the wear can be determined according to the force–displacement curve. The material properties include the elastic modulus of veins *E*_1_ = 1.12 GPa = 1.12 × 10^3^ MPa = 1.12 × 10^3^ N/mm^2^ = 1.12 kN/mm^2^, the elastic modulus of membranes *E*_2_ = 1.5 GPa = 1.5 kN/mm^2^, and Poisson's ratio *υ* = 0.25.

It is difficult to set up accurate 3-dimensional finite element models, because the cross-section corrugation is very small relative to the area of membranous wings of Chinese bees and the measuring accuracy of experimental instruments is very limited. The distributions and structure features of the veins and membranes of membranous wings of Chinese bees are researched emphatically, so establishing 2-dimensional finite element models are enough.

**Figure 4:**
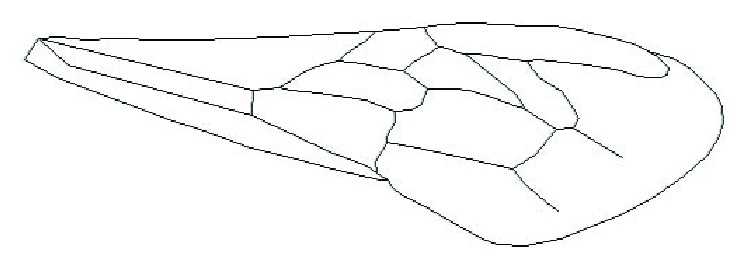
Vein distribution of the right forewing of a Chinese bee.

The structure model of membranous wings of Chinese bees made with ImageWare was imported into AutoCAD. The veins were drawn according to the geometric characteristics and distributions of veins of membranous wings of Chinese bees, as shown in Figure 4.

**Figure 5:**
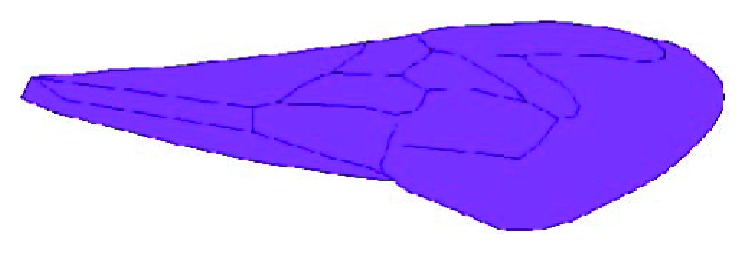
Finite element model of the right forewing of a Chinese bee.

The veins of membranous wings of Chinese bees were turned into surfaces and then imported into ANSYS. The membranes were added on the model. Due to characteristic parameters of membranous wings of Chinese bees, the element type, material property, real constant, and element attribute of membranous wings were defined and the complete finite element model was produced, as shown in Figure 5.

**Figure 6:**
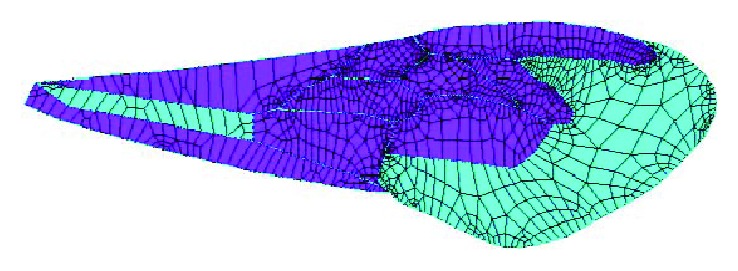
Finite element model meshed, and different colors show different element normals.

The finite element model of membranous wings of Chinese bees was meshed. The finite element model can be divided into quadrilateral meshes or triangular meshes, and computational accuracy of quadrilateral meshes is higher than triangular meshes, so the quadrilateral meshes were adopted. The dimensions of meshes were set and local meshes refined.  Figure 6 shows the finite element model meshed, and different colors show different element normals.

## 3. Results and Discussion

The flight mode of Chinese bees is flapping wing flight. The membranous wings of Chinese bees bear various loads in flight but fit well and fly steadily, and the damage or deformation is slight. Aiming at the flight characteristics of membranous wings of Chinese bees, several essential stress states were simplified. The deformations and stress states of the finite element model were researched under the concentrated force, uniform load, and torque.

### 3.1. Nanomechanical Behaviour under Concentrated Force

The concentrated force was imposed on the finite element model of membranous wings of Chinese bees. The base of the finite element model was fixed, and the concentrated force was exerted on the rightmost node. The concentrated force is the ratio of gravity of the Chinese bee to areas of its all-membranous wings. The areas of forewings of the Chinese bee are larger than those of hindwings, and the ratio is about 3 to 2. The average mass of Chinese bees is about 94.38 mg measured by the electronic analytical balance above. The concentrated force born by the right forewing should be three tenths of the gravity of the Chinese bee, namely, 2.83 × 10^−7^ kN.

**Figure 7:**
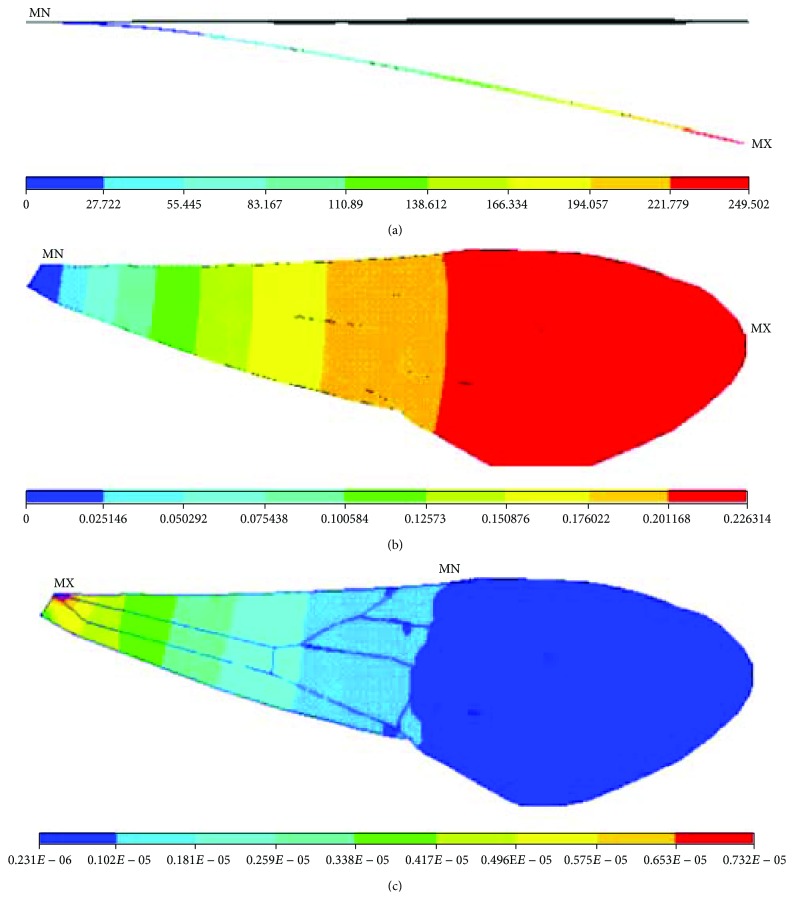
Nanomechanical behaviours of the finite element model of the right forewing of a Chinese bee under concentrated force: (a) displacements; (b) rotation angles; (c) stresses.

In order to observe clearly, the displacements of finite element models were magnified 200 times, and the rotation angles and stresses distributions remained unchanged.  Figure 7 shows the nanomechanical behaviours of finite element models of membranous wings of Chinese bees under concentrated force. The deformations of finite element models increase from the basal part to the end gradually. The maximum structural deformations appear on the end of the models. The maximal displacement is 1.25 mm and maximal rotation angle 0.226 rad, which illustrates the deformations of membranous wings of Chinese bees being large in flight. The large deformations are mainly because there are less veins and more membranes on the membranous wings of Chinese bees and the stiffness of membranes is far less than that of the veins. It is shown that the whole model deforms evenly, and the parts deforming abnormally do not appear. It is indicated that the veins and membranes combine well into a whole to transmit loads effectively and the capacity of bearing load and resisting deformation of the membranous wings is increased. The stresses of finite element models decrease uniformly similar to displacements and rotation angles. The maximal stress appears on the top of the basal part and the minimal stress on the upper edge of the middle part.

### 3.2. Nanomechanical Behaviour under Uniform Load

The uniform load is one of the most important loads borne by insect membranous wings in flight. The uniform loads were exerted on the main veins and the whole membranous wings of Chinese bees, and the deformations and stress states of the finite element model were investigated.

**Figure 8:**
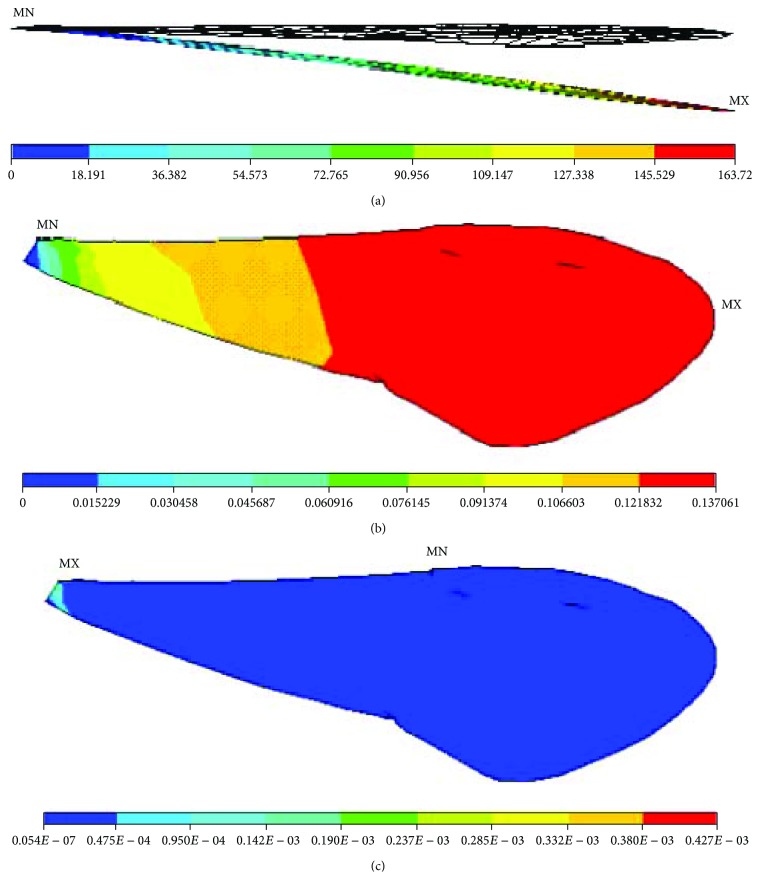
Nanomechanical behaviours of the finite element model of the right forewing of a Chinese bee under uniform load exerted on the anterior longitudinal vein: (a) displacements; (b) rotation angles; (c) stresses.

The concentrated force borne by the right forewings of Chinese bees was scattered on the principal anterior longitudinal vein homogeneously. The uniform load is the ratio of concentrated force to the length of right forewing and is imposed on the finite element model vertically. The concentrated force borne by the right forewing is 2.83 × 10^−7^ kN calculated above and the mean length of the right forewing is about 8.6 mm surveyed with the image analysis system above, so the uniform load is 3.3 × 10^−8^ GPa.  Figure 8 shows the nanomechanical behaviours of finite element models of membranous wings of Chinese bees under uniform load exerted on the anterior longitudinal vein. It is illustrated that the changing trends of displacements and rotation angles under uniform load are similar to those under concentrated force but stress distributions are different obviously. The displacements are smaller than those under concentrated force. Due to the uniform load exerted on the principal anterior longitudinal vein, the rotation angles on the upper edge of the finite element model are greater than those on the lower edge and the maximal rotation angle is smaller than that under concentrated force. The stresses are mainly focused on the basal part of the finite element model and small on the rest.

**Figure 9:**
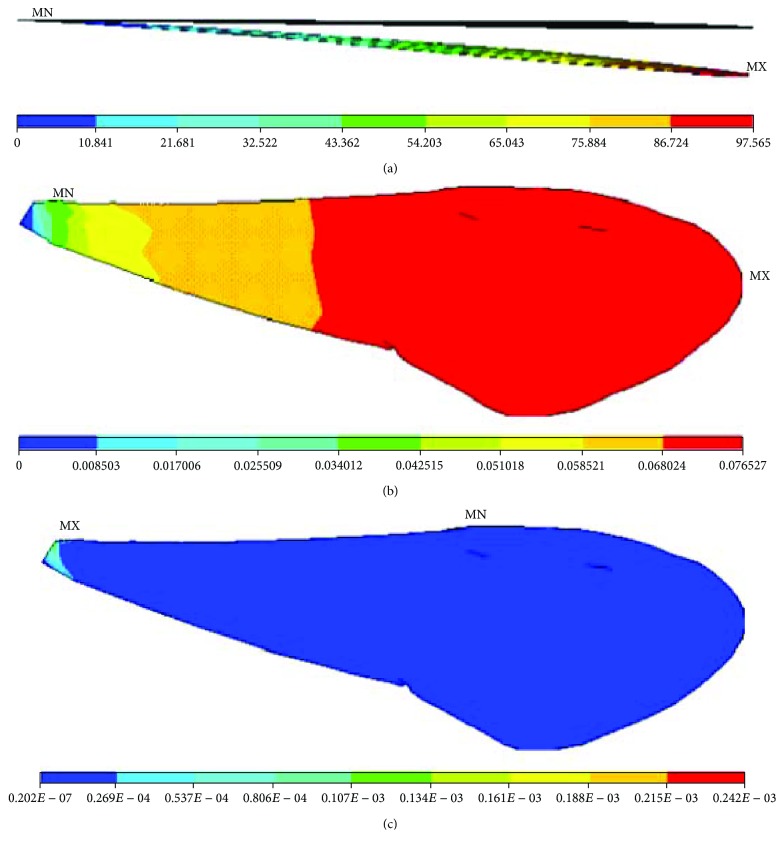
Nanomechanical behaviours of the finite element model of the right forewing of a Chinese bee under uniform load exerted on the whole membranous wings: (a) displacements; (b) rotation angles; (c) stresses.

The uniform loads were imposed on the whole membranous wings of Chinese bees perpendicularly, and the magnitude is the ratio of concentrated force borne by the right forewing to its area. The average area of the right forewing is about 13.2 mm^2^ tested with the image analysis system above, so the uniform load computed is 2.14 × 10^−8^ GPa. The restraint was exerted on the basal part of the finite element model.  Figure 9 shows the nanomechanical behaviours of finite element models of membranous wings of Chinese bees under uniform load exerted on the whole membranous wings. It is shown that the changing trends are alike to those under uniform load exerted on the anterior longitudinal vein, but the displacements and stresses are both reduced. The stresses are still mainly focused on the basal part of the finite element model and distribute on the whole membranous wings uniformly.

### 3.3. Nanomechanical Behaviour under Torque

**Figure 10:**
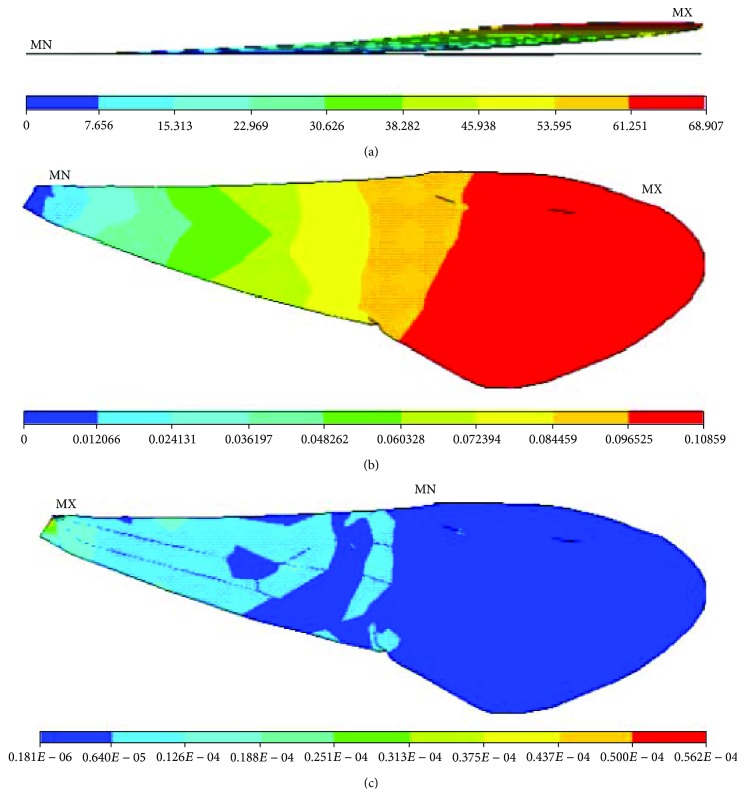
Nanomechanical behaviours of the finite element model of the right forewing of a Chinese bee under torque: (a) displacements; (b) rotation angles; (c) stresses.

A pair of loads whose magnitudes are equal and directions contrary was exerted on the leading edge and trailing edge of finite element models of membranous wings of Chinese bees vertically. The magnitude is 2.14 × 10^−8^ GPa equal to the uniform load exerted on the whole membranous wings above. The displacement constraint was exerted on the basal part of the finite element model, the model was fixed, and then a pair of loads was imposed.  Figure 10 shows the nanomechanical behaviours of finite element models of membranous wings of Chinese bees under torque. It is shown that only overall deformation of the model occurred under torque and the magnitude is far less than that under uniform load exerted on the whole membranous wings. It is illustrated that the model deviates upward from the initial position and the maximal displacement is 0.34 mm appearing on the end of the finite element model. Under the same loads, the large deformations are primarily due to only less and slender veins distributing on the trailing edge of membranous wings of Chinese bees. It is illustrated that rotation angles of finite element models increase from the basal part to the end little by little. On the same cross section, the deformations on middle parts are less than those on the leading edge and trailing edge, and the maximal rotation angle is 0.11 rad. There are no unusual deformations arising, and the deformations are small and transform uniformly, which once again testifies to the membranous wings of Chinese bees having fine global mechanical behaviour and structure stiffness.

It is indicated that the stress distributions of finite element models under torque are unlike those under concentrated force or uniform load. The greater stresses do not focus on the basal part of the finite element model totally and decrease from the basal part to the middle part gradually. The smaller stresses concentrate on the middle and posterior parts, and the maximal stress is 0.56 × 10^−4^ GPa arising on the basal part of the finite element model. On the same cross section, the stresses on the leading edge are larger than those on the trailing edge, which is due to the strong veins with great rigidity, and small deformation can bring large stresses under the same loads.

## 4. Conclusions

The surfaces of membranous wings of Chinese bees were scanned with a 3D laser-scanning system, and the systematic point cloud data were obtained. The defective points and data unrelated to research were deleted, and then the point cloud data were smoothed and simplified with reverse engineering. The 3-dimensional geometry model was rebuilt according to the appearance characters of membranous wings of Chinese bees. The deformations and stress states of the finite element model of membranous wings of Chinese bees were researched under the concentrated force, uniform load, and torque. The deformations of finite element models increase from the basal part to the end gradually and the maximum structural deformations appear on the end of models under concentrated force. The stresses decrease uniformly similar to displacements and rotation angles, and the maximal stress appears on the top of the basal part and minimal stress on the upper edge of the middle part. The displacements and stresses under uniform load exerted on the anterior longitudinal vein are smaller than those under concentrated force. The stresses are mainly focused on the basal parts of models and small on the rest. The changing trends under uniform load exerted on the whole membranous wings are alike to those under uniform load exerted on the anterior longitudinal vein, but the displacements and stresses are both reduced. The large displacement appears on the end of models under torque. The rotation angles increase from the basal part to the end little by little, and on the same cross section, the deformations on the middle part are less than those on the leading edge and trailing edge. The greater stresses decrease from the basal part to the middle part gradually, and the smaller stresses concentrate on the middle and posterior parts. It is indicated that the veins and membranes combine well into a whole, and the deformations are small and transform uniformly, which illustrates the membranous wings of Chinese bees having excellent mechanical behaviour and structure stiffness.
